# Molecularly Imprinted Composite Membranes for Selective Detection of 2-Deoxyadenosine in Urine Samples

**DOI:** 10.3390/ijms160613746

**Published:** 2015-06-16

**Authors:** Sonia Scorrano, Lucia Mergola, Maria Pia Di Bello, Maria Rosaria Lazzoi, Giuseppe Vasapollo, Roberta Del Sole

**Affiliations:** Dipartimento di Ingegneria dell’Innovazione, Università del Salento, via per Arnesano Km 1, Lecce 73100, Italy; E-Mails: lucia.mergola@unisalento.it (L.M.); mariapia.dibello@unisalento.it (M.P.D.B.); mariarosaria.lazzoi@unisalento.it (M.R.L.); giuseppe.vasapollo@unisalento.it (G.V.); roberta.delsole@unisalento.it (R.D.S.)

**Keywords:** 2-deoxyadenosine, molecularly imprinted membrane (MIM), urine detection, selective separation

## Abstract

An important challenge for scientific research is the production of artificial systems able to mimic the recognition mechanisms occurring at the molecular level in living systems. A valid contribution in this direction resulted from the development of molecular imprinting. In this work, a novel molecularly imprinted polymer composite membrane (MIM) was synthesized and employed for the selective detection in urine samples of 2-deoxyadenosine (2-dA), an important tumoral marker. By thermal polymerization, the 2-dA-MIM was cross-linked on the surface of a polyvinylidene-difluoride (PVDF) membrane. By characterization techniques, the linking of the imprinted polymer on the surface of the membrane was found. Batch-wise guest binding experiments confirmed the absorption capacity of the synthesized membrane towards the template molecule. Subsequently, a time-course of 2-dA retention on membrane was performed and the best minimum time (30 min) to bind the molecule was established. HPLC analysis was also performed to carry out a rapid detection of target molecule in urine sample with a recovery capacity of 85%. The experiments indicated that the MIM was highly selective and can be used for revealing the presence of 2-dA in urine samples.

## 1. Introduction

A great interest in membranes has been increasing in various fields of science and technology because membranes play an indispensable role in the solving of basic problems such as in the fields of resource, energy, and information [1,2,3]. A wide range of microfiltration, ultrafiltration, nanofiltration, and reverse osmosis membranes is now available and used for an increasing number of separation applications in industry, environmental protection, life sciences, medicine and biotechnology [4]. In addition, membrane separation has attracted much attention owing to its promising properties such as easy and low energy continuous operation under mild conditions; better feasibility to scale-up; and the resulting low cost of operation [5]. Typical commercial membranes do not allow selective separation of individual substances, so much effort has been made to develop new membranes with controlled specificity for individual compounds [6,7].

In the last few years, the preparation of novel membranes using stable Molecularly Imprinted Polymers (MIPs) having a specific synthetic receptor structure resulted in an attractive method that might be a possible solution for this challenge. Molecular imprinting is the most applicable method for the introduction of molecular recognition properties in synthetic polymers in response of the presence of template species during formation of the three-dimensional structure of the highly cross-linked polymer [8,9]. However, new MIP formats are being developed to avoid the limitations of the traditional approach: long preparation times, mechanical deformation of the binding sites during grinding of bulk polymers, and a time-consuming sieving procedure. To solve most of these problems, several research groups attempted the fabrication of patterned (or corrugated) membranes for various applications. Molecularly Imprinted Membranes (MIMs) offer the advantage of combining the mechanical integrity of the support membrane and the selectivity of the imprinted polymer to separate the template specie from a mixture of compounds present in solution by permeating through the thin membranes [10]. Compared with MIPs prepared by conventional methods, MIMs have notable advantages such as large specific surfaces, providing relatively high imprinting sites per unit mass, and fine porous structures, resulting in accessibility of imprinting sites and the low diffusion resistance necessary for high efficiency and easy recoverability from practical operation or applicability for continuous usages [11,12]. Moreover, the MIMs can be achieved by immobilizing the imprint layer onto the substrate membrane without destroying the specific recognition sites during the grinding step [13,14]. Besides, the substrate can endow the imprint membrane with robust and self-supporting properties. After the first application of MIP to membrane separation [15], various methods were reported to prepare MIMs, such as: *in situ* polymerization by thermal or UV initiated bulk cross-linking [16,17], surface imprinting [18,19], dry or wet phase separation [20,21], incorporation of molecularly imprinted nano-spheres or particles to obtain composite membrane [22,23]. MIP membranes are always prepared as thin polymer films on the surface of support membranes or as freestanding membranes, either from previously synthesized conventional MIPs or from the simultaneous formation of an MIP structure with membrane morphology. Generally, cellulose acetate, nylon, polyvinylidene-difluoride (PVDF) were used as supporting membranes. Indeed, PVDF membrane is an ideal support due to its excellent chemical and thermal stability, mechanical strength and filtration performance; furthermore, the porous structure of PVDF membrane with high flux optimized by a phase inversion method, is in favor of the immobilization of imprint layer.

Starting from our experience in the field of molecularly imprinted polymers for the detection of disease biomarkers [24,25], in this work the surface imprinting technology was used to study and develop more simply and quickly a new material selective for a rapid detection in urine samples of a specific tumoral biomarker such as 2-deoxyadenosine (2-dA). The total number of modified nucleosides in various types of RNA was found to be 93 [26], of which more than 53 have been identified in urine, and, of those, 14 are adenosine derivatives [27]. Enzymatic modification processes like methylation, hydroxylation, reduction, isomerization and addition of complex side chains proceed post-transcriptionally at the macromolecular level [28]. It is known that patients suffering from cancer diseases excrete with their urine increased amounts of modified nucleosides, including 2-dA. Different diagnostic tools may be used to qualitatively analyze and quantify these markers, such as antibody-based assays or other analytical techniques (e.g., LC–MS) [29,30,31,32,33,34]. However, most of these procedures are quite laborious and the selective analysis of the urinary nucleosides could greatly simplify the analysis of the urine samples. So, the aim of this study was to achieve the specific adsorption of urinary nucleosides by the application of molecularly imprinted membranes. By thermal polymerization, the 2-dA-MIM was cross-linked on the surface of the PVDF support and, after characterization, its binding affinity and selectivity were tested in batch experiments. A corresponding non-imprinted membrane (NIM), prepared using the same procedure in absence of 2-dA, and an original PVDF membrane (blank) were tested to demonstrate the absence of binding affinity. HPLC analysis was also performed to carry out a rapid detection of molecule target in urine sample. The experiments indicated that the MIM was highly selective and can be used to reveal the presence of 2-dA in urine samples.

## 2. Results and Discussion

### 2.1. Preparation of Molecularly Imprinted Membranes

The hydrophilic PVDF membrane was selected as supporting membrane. In order to remove residual substances and activate the hydrophilic functional groups on the membrane surface, different membrane conditioning methods were tried, including immersion in pure water, acetonitrile and AIBN-acetonitrile solution. It was found that, when the PVDF membrane was soaked in AIBN solution, the next cross-linked reaction was favored. Thus, in the preparation of MIMs, the support membrane was dipped into the imprinting solution, where the polymerization coating occurred not only on the external surfaces but also in the internal pores of membranes. It was also of great importance to select an appropriate immersion time in polymerization solution for the PVDF membrane to obtain a good pre-polymerization support. In accordance with the literature synthesis procedures [35,36], it was found that the optimal polymerization time, at 60 °C, was 46 h. By varying the pre-polymerization and polymerization time, MIMs with modification degree (*D*_m_) of 2.40 mg/cm^2^ were prepared, while the *D*_m_ for the non-imprinted membranes was 2.52 mg/cm^2^. Using shorter polymerization times, lower *D*_m_, associated with a limited repeatability, were measured This indicates that MIMs and NIMs have the similar thickness of the coating layer and the modest difference may come from the washing procedure, after which the recognition cavities and molecular paths are formed in the imprinted layer [37]. Moreover, the imprinted membranes prepared under the same procedure showed a uniform behavior after binding experiment.

### 2.2. Characterization of the Membranes

**Figure 1:**
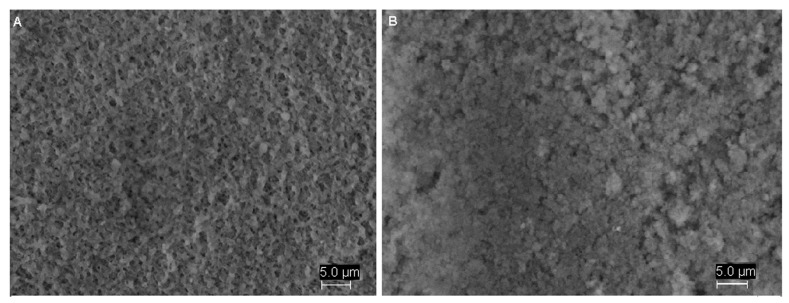
SEM images of the PVDF membrane (**A**) and MIM (**B**).

To prove the nature of the imprinting process on the membrane, Scanning Electron Microscopy (SEM), Energy-Dispersive X-ray Spectroscopy (EDS) and Fourier Transform Infrared Spectroscopy (FTIR) analyses were performed. SEM was used to study the physical and morphological characteristics, in terms of pore size and porosity, of the membranes. There are substantial differences in morphology between the MIM and an original PVDF membrane (Figure 1). It can be seen that the membrane surface becomes more smooth and uniform after modification. Moreover, the blank PVDF membrane had a medium porous fibrous structure (Figure 1A). When polymerization was performed on this supporting membrane, its surface morphology was greatly altered and the number of pores decreased when the polymer layer was formed on the surface of the PVDF membrane (Figure 1B). It can be hypothesized that the top surface of the PVDF membrane was modified after polymerization process. The pore size was determined with SEM image analysis using the ImageJ software. The most frequent pore diameter found was 0.250 ± 0.042 µm for the virgin membrane and 0.150 ± 0.006 µm for the MIM. These results revealed that the pore size of PVDF membrane decreases after polymerization. Further analyses were needed to prove the efficiency of the imprinting effect.

**Figure 2:**
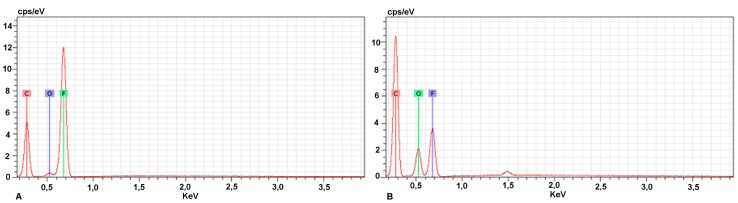
EDX spectra of PVDF membrane (**A**) and MIM (**B**).

Surface chemistry changes can be verified from EDX and FTIR results. A quantitative EDX analysis was performed to establish the difference in main chemical elements between the original and the imprinted membrane. These elements were identified as carbon and fluorine atoms originating from the PVDF structure while the increase in C and O is clearly highlighted with the imprinted membrane compared to the virgin one. In Figure 2, the EDX spectra of the PVDF membrane (A) and the MIM (B) are reported.

**Figure 3:**
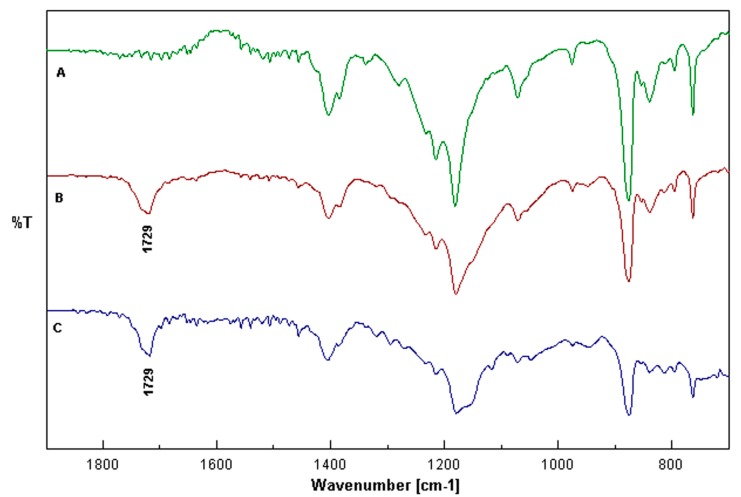
FTIR spectra of a blank PVDF membrane (A), PVDF + NIM (B) and PVDF + MIM (C).

As expected, the peak associated to the fluorine atom is more intense in the original membrane (Figure 2A), while the fluorine peak decreased for the membrane after polymerization (Figure 2B). The percentage of fluorine resulted 44% on the surface of the original PVDF membrane and 18% on the surface of the MIM, with respect to the components of the substrate. Elemental analysis also showed a significant increase in the percentage of C and O atoms in the MIM compared with the blank membrane. Indeed, the percentage of carbon and oxygen resulted 52% and 3%, respectively, in blank membrane and 65% and 17% on the surface of the MIM. Moreover, a semi-quantitative relationship of the chemical elements can be calculated based on the peak sizes in the spectrum. These differences confirm the presence of a polymerization layer on PVDF surface. This issue can be clarified by Fourier Transform Infrared technique. The FTIR spectra of original, PVDF-MIM and PVDF-NIM membranes are shown in Figure 3.

The absorption peaks at 1403, 1072, 880 and 840 cm^−1^ are the typical signals of PVDF. All three samples revealed the C–F vibrations at 1215 and 1180 cm^−1^ [38]. The most significant change in the spectra of the modified membranes (Figure 3B,C) is a typical peak at 1729 cm^−1^, which is not present in the spectrum for the blank membrane (Figure 3A). This peak is due to the presence of C=O bond stretching in carboxylic acid and ester groups and indicates that the pre-modification of the PVDF membranes is performed via synthesis of a cross-linked hydrophilic polymer covering the surface of the porous membrane [39]. Both surface chemistry and morphology changes have an impact to the final performance of the imprinted composite membranes.

### 2.3. Adsorption Kinetic

**Figure 4:**
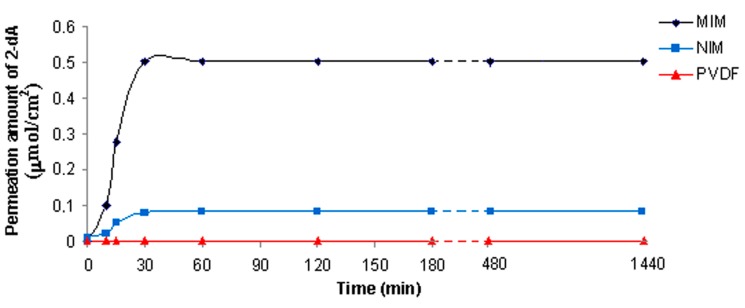
Kinetic study for 2-dA sorption of MIM, NIM and PVDF membrane.

The kinetic of the sorption process is an important parameter in evaluating membrane quality and the detection efficiency. The MIM, NIM and PVDF membrane sorption capacity of 2-deoxyadenosine were measured as a function of time. Figure 4 shows the adsorption kinetic curves of 2-dA on membranes in MeCN/H_2_O solution containing 0.5 mmol/L of 2-dA *versus* different adsorption time.

As it can be seen, in the initial step, the adsorption rate of 2-dA for MIM was faster than other membranes; in particular, a rapid increase in molecule sorption was observed during the first 30 min, reaching equilibrium state with an adsorption efficiency of the template of 0.5 µmol/cm^2^. The fast adsorption at the initial stage, not found in NIM and the virgin membrane, may be due to the availability of the active sites on the 2-dA-MIM surface area as a result of imprinting effect. Hence, 2-dA molecules can reach the imprint sites easily and quickly during the rebinding step. Therefore, the membrane showed good site accessibility for template molecule and equilibrium was achieved quickly. After 30 min, there is no significant difference in adsorption. This behavior could be due to a rapid saturation of the imprinted sites for 2-dA. Instead, the MIP particles prepared by the conventional bulk technique require about 20 h to reach the equilibrium [24,25]. Moreover, the advantage of the imprinted membrane prepared in this work was given to the rapid rate of binding compared to similar membrane systems where the sorption kinetics reached equilibrium at 150 or 180 min [40,41]. As a conclusion, higher adsorption efficiency was obtained in a shorter time for molecule detection with the MIM, making it a suitable system that is less laborious and quicker than other diagnostic tools for rapid 2-dA detection.

### 2.4. Binding Capacities of the MIMs

**Figure 5:**
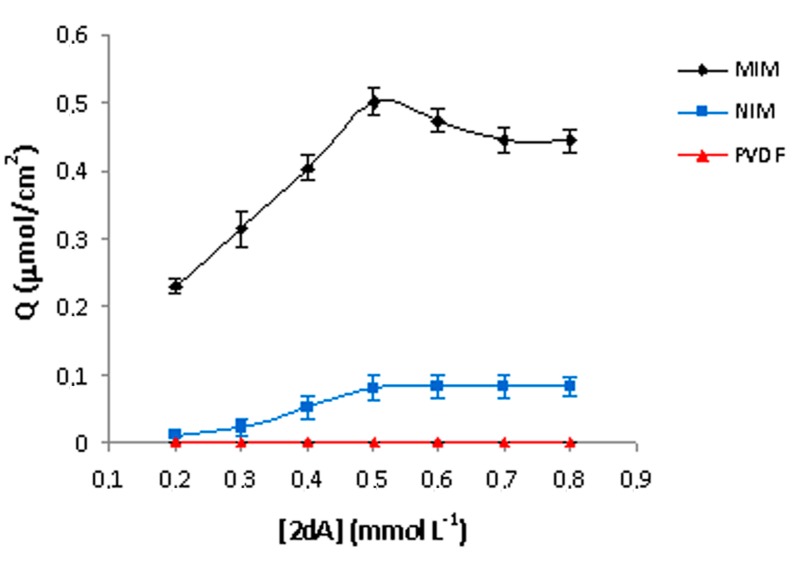
Batch rebinding experiments of MIMs, NIMs and PVDF membranes.

In order to further understand the binding capacity of 2-deoxyadenosine in membranes, binding isotherms were carried out. The binding curves reported in Figure 5 show the amount of template bound per cm^2^ (*Q*) of MIM, NIM and PVDF membranes in 30 min, as a function of the initial concentration of 2-dA ranging from 0.2 to 0.8 mmol·L^−1^.

**Figure 6:**
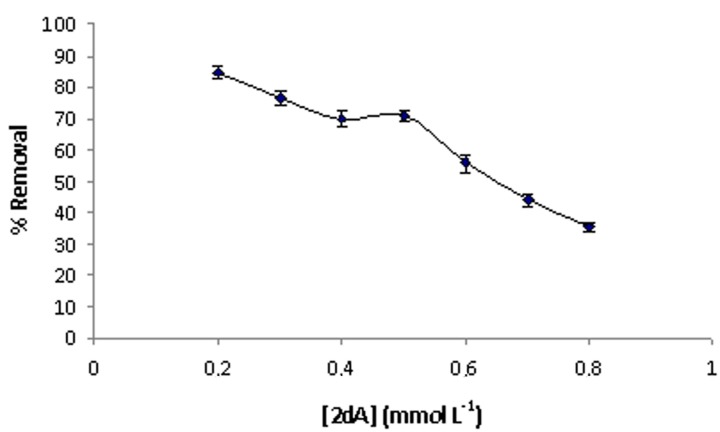
Percent removal of 2-dA by the MIM.

MIM curve shows an initial increase in binding, followed by saturation at the concentration value of 0.5 mmol·L^−1^, indicating that the available receptor sites have been saturated with 2-dA. Moreover, as can be seen, the MIM had higher binding capacity than non-imprinted membrane, due to the recognition cavities formed in the imprinted layer of MIM. On the contrary, the blank membrane showed no binding capabilities towards 2-dA. Batch binding results for MIM are processed as a percentage of removal of the 2-dA according to Equation (3) (Figure 6). It can be seen that the percentage of removal decreases from 84% to 27% with the increase of the template dosage from 0.2 to 0.8 mmol·L^−1^. The decrease of 2-dA adsorption is due to the fact that available receptor sites have been saturated with template in the system.

**Figure 7:**
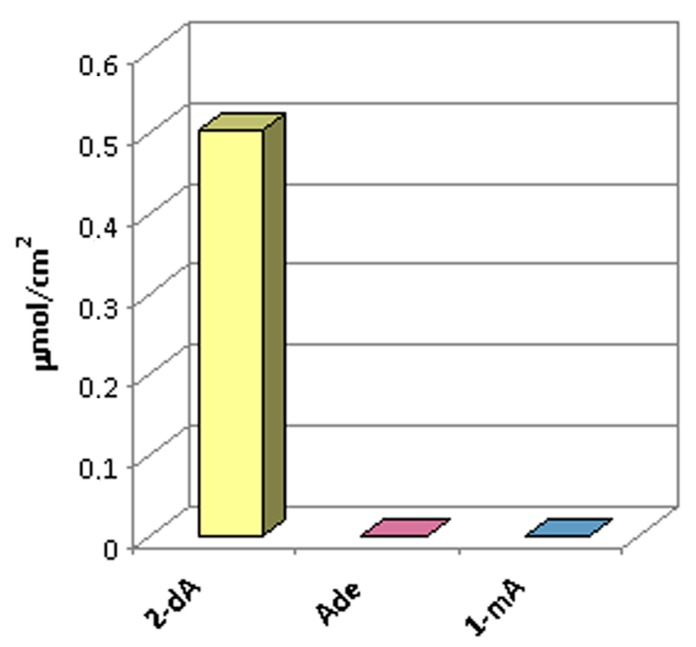
Selectivity studies of MIM using a mixture solution of 2-deoxyadenosine, adenosine and 1-methyladenosine at a concentration of 0.5 mmol·L^−1^ each.

To investigate the target selectivity, the saturated binding amounts of imprinted membrane towards different analytes were evaluated. In particular, two other structurally related modified nucleosides (adenosine and 1-methyladenosine) were chosen. Similar to batch rebinding experiments for 2-dA, selectivity study were also conducted at the concentration of binding capacity saturated (0.5 mmol·L^−1^) in MeCN/H_2_O solution. As shown in Figure 7, the 2-dA imprinted membrane exhibited relatively high binding affinities for 2-dA, while it did not show any binding capacity towards the other compounds. The reason for this is that the MIM can recognize its template molecule due to the existence of memory cavities of fixed size and shape, binding sites, and specific binding interactions between the target molecule and sites [35]. Also as expected, the non-imprinted (NIM) and the blank PVDF membranes did not show any binding capacity for these nucleosides.

### 2.5. Urine Sample Analysis

**Figure 8:**
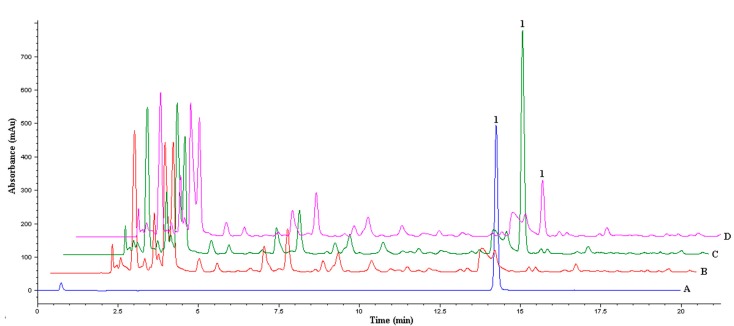
Chromatograms corresponding to (A) 2-deoxyadenosine in H_2_O; (B) urine sample; (C) urine spiked with 2-dA; and (D) urine after MIM incubation with spiked-urine. Peak identification 1: 2-deoxyadenosine (2-dA).

The prepared MIM was applied as detection material for analysis of a real sample. The chromatograms obtained from urine samples are displayed in Figure 8.

Compared to the chromatogram (C) of urine spiked with 2-dA, the chromatogram (D) was obtained after MIM incubation and it was demonstrated that the MIM can retain trace amounts of 2-dA. The MIM recovery capacity of 2-dA in the spiked samples resulted 85% with RSD lower than 2%. These results indicated that MIM could be used directly as efficient material for determining real samples.

## 3. Experimental Section

### 3.1. Reagents and Apparatus

2-Deoxyadenosine (2-dA), adenosine (ade), 1-methyladenosine (1-mA), ethylene glycol dimethacrylate (EGDMA), acetic acid and polyvinylidene fluoride (PVDF) Millipore membranes with a 0.22 μm nominal pore size and 13 mm in diameter were purchased from Sigma–Aldrich (Steinheim, Germany). Methacrilylic acid (MAA) and α,αʹ-azoisobutyronitrile (AIBN) were supplied from Fluka (Steinheim, Germany). Analytical grade acetonitrile (MeCN) and methanol (MeOH) were obtained from J.T. Baker (Deventer, The Netherlands). Deionized water was provided by a water purification system (Human Corporation, Seoul, Korea). Batch rebinding experiments were carried out using a Cary 100 Scan UV–visible spectrophotometer (Varian, Palo Alto, CA, USA). Sonication was carried out using a Sonorex RK 102H ultrasonic water bath from Bandelin Electronic (Berlin, Germany). Centrifugation was achieved with a PK121 multispeed centrifuge from Thermo Electron Corporation (Chateau-Gontier, France). SEM images were recorded with a Zeiss EVO 40 microscope (Jena, Germany), at an accelerating voltage of 20 kV. MIM and blank PVDF membrane were analyzed (at magnification 5000×) to record the morphology of the membrane surface. Elemental analyses were performed using the SEM equipped with EDS analysis (Bruker 127 eV mod. XFlash detector 5010; Bruker, Berlin, Germany). FTIR analysis were recorded on a JASCO 660 plus infrared spectrometer (Jasco, Gross-Umstadt, Germany) placing the membranes directly on an ATR ZnSe crystal to characterize the preparation of imprinted layer on substrate. The absorption spectra of membranes were recorded in the wavenumber range of 4000–600 cm^−1^ by cumulating 16 scans at a resolution of 4 cm^−1^. HPLC analyses were performed using an Agilent 1100 Series LC/MSD system coupled to a photo-diode array detector. Chromatography separation was carried out on a 150 × 4.6 mm i.d., 5 µm SS Wakosil C18 column with a 4 × 3 mm i.d. Phenomenex C18 guard cartridge, both thermostatted at 25 °C. The mobile phase was composed of 5 mM sodium acetate buffer (pH 5) (solvent A) and acetonitrile (solvent B) at a flow rate of 0.8 mL·min^−1^. The following gradient was utilized: 0 min, 2% B; 8 min, 5% B; 13 min, 10% B; 18 min, 20% B; and 20 min STOP. The chromatograms were acquired at the wavelength of 259 nm.

### 3.2. Preparation of Molecularly Imprinted Membrane

Circular hydrophilic PVDF filters (area = 1.3 cm^2^) were activated as following: membranes were rinsed first with pure water and subsequently with MeCN, and then soaked in a 0.15 mol/L AIBN acetonitrile solution for 15 min. Finally membranes were taken out and dried under vacuum. The activated membranes were quickly soaked in the mixture of molecular imprinting solution.

The 2-dA-MIM was prepared by thermal polymerization using 2-deoxyadenosine as template. 2-dA (0.0234 mmol) was dissolved in 1 mL of acetonitrile/water (4/1 *v*/*v*) in a glass tube for 5 min. Next, MAA (0.33 mmol) and EGDMA (1.76 mmol) were added to the solution and, after 5 min, AIBN (0.02 mmol) was added and dissolved in an ultrasonic bath under nitrogen gas for 5 min to remove oxygen. After that, the PVDF membrane was immersed in the solution and left to initiate thermally the polymerization at 60 °C for 30 min. Then, the membrane was removed and clamped with two glass plates pressing until air bubbles were removed completely. The plates were sealed and heated in an oven at 60 °C to continue the polymerization for 46 h. The membrane was then washed with methanol/acetic acid (7:3 *v*/*v*) to extract the template and finally with methanol to eliminate the residual acid. The efficiency of washing procedure was checked by recording of the UV spectrum of the filtrate to guarantee that the absorbance was less than 0.005 at 259 nm. The extracted membrane was finally dried to constant weight at 60 °C and the degree of modification (*D*_m_) was calculated as follows:

*D*_m_ = (*m*_c_ − *m*_o_)/*m*_o_ (1)

where *m*_o_ and *m*_c_ are the dry weight of original and modified membrane after extraction.

For comparison, the reference composite non-imprinted membrane (NIM) was prepared under the same polymerization procedure but in the absence of any template. Different imprinted membranes were prepared with the same procedure and were kept in a desiccator for use.

### 3.3. Kinetic Adsorption Test

The kinetic adsorption experiment was conducted as follows: MIM was immersed into 2 mL of 2-dA in MeCN/H_2_O (4:1 *v*/*v*) solution at known concentration (0.5 mM) and kept under constant stirring. Aliquots of the solution (10 µL) were taken at time intervals of 5, 10, 15, 30, 60 min until 24 h and the concentration of unbound compounds at the different absorption time was measured by UV–vis spectrophotometer at 259 nm. The binding amount of 2-dA on MIM was determined by the difference between the total 2-dA amount and residual amount of the solution. The optimum time for 2-deoxyadenosine recognition using the synthesized MIM was evaluated.

### 3.4. Binding Experiments of 2-dA on Membranes

The capacity of the MIMs to selectively rebind the template molecule 2-dA, was analyzed by batch rebinding experiments The 2-dA-MIMs, NIMs and the PVDF membrane were immersed in 2 mL of MeCN/H_2_O (4:1 *v*/*v*) solution spanning the concentration range from 0.2 to 0.8 mM of 2-deoxyadenosine, at room temperature for 30 min under constant stirring. The resulting solution after incubation was analyzed at 259 nm. The amount of 2-dA bound to the MIM was calculated according to the change of molecule concentration in solution before and after the binding experiments. The 2-dA uptake on membrane defined as adsorption capacity, *Q* (µmol/cm^2^) and the percentage of removal, % removal, were calculated by using Equations (2) and (3), respectively:

*Q* = *V*(*c*_0_ − *c*)/*A*_m_ (2)

where *c*_0_ (µmol/mL) and *c* (µmol/mL) are the initial and equilibrium concentrations of 2-dA in solution, respectively; *V* (mL) is the total volume of the solution; and *A*_m_ (cm^2^) is the area of the membrane.

% Removal = (*c*_0_ − *c*)/*c*_0_ × 100 (3)

where *c*_0_ and *c* are the initial and equilibrium concentrations of 2-dA. Data were acquired in replicates of three with RSD < 5%.

In order to verify the selectivity of the MIMs, the binding of other RNA nucleosides, which are adenosine and 1-methyadenosine on the polymers, was investigated.

### 3.5. Extraction of 2-dA from Spiked Human Urine

To determinate the effectiveness of detection of the imprinted membrane, the MIM prepared as described above, was immersed in an aliquot (2 mL) of human urine spiked with 2-dA (0.5 mM) and incubated for 30 min at room temperature under stirring. One milliliter of supernatant was removed for analysis by HPLC. The sample before and after incubation was analyzed and the concentration of 2-deoxyadenosine bound to the MIM was calculated. The calibration curve in the concentration range of 0.5–0.1 mmol·L^−1^ was constructed from the peak area *versus* 2-deoxyadenosine concentration. The obtained regression equation showed good linearity with a correlation coefficient *R*^2^ of about 0.9995. The experiments were repeated three times.

## 4. Conclusions

This study described the synthesis of a cross-linked imprinted membrane having a strong affinity for polar compounds such as nucleosides. In particular, a highly selective MIM for 2-deoxyadenosine was prepared and its affinity to bind target molecule was confirmed as a result of imprinting effect. The selective properties of the obtained membrane were evaluated and its use as detection material for 2-dA from urine was demonstrated. Moreover, SEM, EDX and FTIR analyses were used to study the surface morphological and chemical characteristics of the synthesized 2-dA-MIM. This work demonstrates the feasibility of using this membrane for the selective recognition of 2-dA from real urine samples and it will contribute to the development of new systems, based on imprinted polymer, for early monitoring of cancer biomarkers in biological fluids.
